# Analysis of the components of cancer risk perception and links with intention and behaviour: A UK-based study

**DOI:** 10.1371/journal.pone.0262197

**Published:** 2022-01-13

**Authors:** Christiane Riedinger, Jackie Campbell, William M. P. Klein, Rebecca A. Ferrer, Juliet A. Usher-Smith

**Affiliations:** 1 Department of Public Health and Primary Care, Prevention Group, Primary Care Unit, University of Cambridge Clinical School, Cambridge, United Kingdom; 2 Faculty of Health, Education and Society, University of Northampton, Northampton, United Kingdom; 3 Behavioral Research Program, National Cancer Institute, Rockville, Maryland, United States of America; Max Planck Institute for Human Development, GERMANY

## Abstract

Risk perception refers to how individuals interpret their susceptibility to threats, and has been hypothesised as an important predictor of intentions and behaviour in many theories of health behaviour change. However, its components, optimal measurement, and effects are not yet fully understood. The TRIRISK model, developed in the US, conceptualises risk perception as deliberative, affective and experiential components. In this study, we aimed to assess the replicability of the TRIRISK model in a UK sample by confirmatory factor analysis (CFA), explore the inherent factor structure of risk perception in the UK sample by exploratory factor analysis (EFA), and assess the associations of EFA-based factors with intentions to change behaviour and subsequent behaviour change. Data were derived from an online randomised controlled trial assessing cancer risk perception using the TRIRISK instrument and intention and lifestyle measures before and after communication of cancer risk. In the CFA analysis, the TRIRISK model of risk perception did not provide a good fit for the UK data. A revised model developed using EFA consisted of two separate “numerical” and “self-reflective” factors of deliberative risk perception, and a third factor combining affective with a subset of experiential items. This model provided a better fit to the data when cross-validated. Using multivariable regression analysis, we found that the self-reflective and affective-experiential factors of the model identified in this study were reliable predictors of intentions to prevent cancer. There were no associations of any of the risk perception factors with behaviour change. This study confirms that risk perception is clearly a multidimensional construct, having identified self-reflective risk perception as a new distinct component with predictive validity for intention. Furthermore, we highlight the practical implications of our findings for the design of interventions incorporating risk perception aimed at behaviour change in the context of cancer prevention.

## Introduction

Risk perception is a common predictor in psychological models of health behaviour change [1–3]. In general, risk perception is theorised to represent the perceived personal likelihood of an adverse event, the emotional and intuitive reactions informed by that likelihood, and consideration of the characteristics and perceived severity of the event [4]. There are consistent reports of an association of risk perception with behaviour in the literature which varies in magnitude [5–7]. Understanding individual interpretations of health threats and how these influence behaviour is therefore important for developing public health interventions involving risk communications.

Originally, risk perception was defined and subsequently measured as a largely deliberative construct, usually on scales capturing perceived likelihood severity [8–11]. As affect has been shown to be an essential component of decision making and risk appraisal [12–14], subsequent operationalisation of risk perception included an affective component, comprising emotional reactions to a threat such as worry, anxiety and fear [15, 16]. In addition, a third proposed category of experiential risk perception relates to heuristic, “gut-level” or intuitive reactions to threat, ostensibly resulting from previously acquired, learned associations. The cognitive basis of experiential risk perception is not considered to be consciously accessible, but there can be awareness of an intuition about how much an individual is at risk of a threat [17]. All three factors of risk perception have been shown to be related to intentions and behaviours [5, 17–20].

The TRIRISK model of risk perception proposed by Ferrer *et al*. [17] was the first to combine the three categories of deliberative, affective and experiential risk perception into a single theoretical model. It was developed and validated in a US population, addressing three different disease conditions: cancer, heart disease and diabetes. The final, validated TRIRISK model included the three categories of risk perception, all of which held together well in psychometric testing across a variety of studies and threats. The original TRIRISK study showed an independent association of each component with protection motivation in relation to cancer. A more recent study replicated the discriminant validity of the model for cancer and selected other illnesses, and confirmed that the predictive ability is enhanced by the characterisation of risk perception as a three-component model [21].

In this study, we aimed to examine the replicability of the TRIRISK model and characterise the inherent factor structure of the TRIRISK risk perception assessment when applied to an online randomised controlled trial investigating the effect of communicating personalised risk of the five most common cancers on risk perception, modifiable lifestyle factors and health behaviour in a UK population [22, 23]. The online surveys of this trial captured the three dimensions of cancer risk perception according to the TRIRISK instrument, as well as information about behavioural intentions and lifestyle changes linked to cancer prevention. The study was carried out in three stages:

**Stage 1**. Using confirmatory factor analysis (CFA), we initially tested the TRIRISK structure of deliberate, affective and experiential risk in the UK dataset. Mirroring the development of the TRIRISK model [17], we assessed whether risk perception was best defined as a single construct or instead a construct composed of two (deliberative and non-deliberative) or three categories (deliberative, affective and experiential).
**Stage 2**. We then performed exploratory factor analysis (EFA) to test if the UK data followed the tripartite structure of the TRIRISK model or displayed a different underlying structure, using a random sub-set of the data for development and the remaining data for cross-validation.
**Stage 3**. Finally, we explored the predictive associations of risk perception factors of Stage 2 with behavioural intention and self-reported behaviour change with respect to cancer prevention.

## Overall study design

Data were taken from an online randomised controlled trial (RCT) investigating the effect of personalised online cancer risk information on risk perception, behavioural intentions, self-reported health behaviours over three months. The trial was hosted by the University of Cambridge. Ethical approval was received prior to the execution of the trial (Psychology Research Ethics committee of the University of Cambridge, Ref: PRE.2017.093). Informed consent was given online. All participants were over 18 years of age.

Full details of this trial have been reported elsewhere [22, 23]. In brief, 1018 participants aged 30 to 73 were recruited to the parent trial using the platform Prolific (https://www.prolific.ac/), and subsequently randomised to one of three intervention groups or a control group. Participants in the intervention groups were given their personalised algorithm-predicted 10-year cancer risk in one of three graphical formats. The questionnaire presented to participants was prepared and delivered using the Gorilla platform (https://gorilla.sc). Participants with previous cancer diagnoses were excluded from the analysis. Data were collected at three time points: prior to risk communication, directly after, and at three months follow up. Stage 1 and 2 analyses in the current study were based on risk perceptions assessed at baseline prior to cancer risk communication, so that we were examining *a priori* beliefs held before the intervention. In the regression analysis of Stage 3, data at baseline, immediate and three-month follow up were used.

Questions addressing risk perception were taken as published from the TRIRISK instrument of risk perception by Ferrer *et al*., consisting of 18 questions relating to three domains (deliberative, affective and experiential) [17]. The Likert response coding was reversed where appropriate to ensure that lower response scores were always associated with lower perception of risk to aid interpretation. Survey details, phrasing of questions and measurement scales are included in Table 1. Questions were presented in numerical order.

**Table 1:** Measures of risk perception.

ID	Question Text	Scale
D1	How likely do you think is it that you will get one of these five cancers at some point in the next 10 years?	1 = Likely to 7 = Unlikely, recoded to 1 = Unlikely to 7 = Likely
D2	On a scale from 0 to 100%, how would you rate the probability that you will develop one of these five cancers in the next 10 years?	Integers of 0–100%, rescaled to 1–7 using Y = X*0.06+1
D3	How do you think your chance of developing one of these five cancers in the next 10 years compares to the average person of your sex and age?	1 = Much lower to 7 = Much higher
D4	The way I look after my health means that my odds of getting one of these five cancers in the future are:	1 = Very low to 7 = Very high
D5	When I think carefully about my lifestyle, it does seem possible that I could get one of these five cancers.	1 = Strongly disagree to 7 = Strongly agree
D6	If I look at myself as if I was a doctor, I realise that my behaviour puts me at risk of getting one of these five cancers.	1 = Strongly disagree to 7 = Strongly agree
A1	How worried are you about developing cancer in the future?	1 = Not at all to 7 = Extremely
A2	How fearful are you about developing cancer in the future?	1 = Not at all to 7 = Extremely
A3	How nervous are you about developing cancer in your lifetime?	1 = Not at all to 7 = Extremely
A4	When you think about cancer for a moment, to what extent do you feel fearful?	1 = Not at all to 7 = Extremely
A5	When you think about cancer for a moment, to what extent do you feel worried?	1 = Not at all to 7 = Extremely
A6	When you think about cancer for a moment, to what extent do you feel anxious?	1 = Not at all to 7 = Extremely
E1	How concerned are you about developing cancer in your lifetime?	1 = Not at all to 7 = Extremely
E2	How easy is it for you to imagine yourself developing cancer in the future?	1 = Not at all to 7 = Extremely
E3	I feel very vulnerable to disease.	1 = Strongly disagree to 7 = Strongly agree
E4	I am confident that I will not get cancer.	1 = Strongly agree to 7 = Strongly disagree
E5	I would be lying if I said “There is no chance of me getting cancer.”	1 = Strongly disagree to 7 = Strongly agree
E6	My first reaction when I hear of someone getting cancer is “that is could be me someday”.	1 = Strongly disagree to 7 = Strongly agree

Abbreviations: D = deliberative, A = affective, E = experiential.

### Stage 1: Confirmatory factor analysis of the TRIRISK model of risk perception using a UK randomised controlled trial

#### Methods

Data analysis was conducted using the statistical program Stata (Version 13, Statacorp, Texas), using the baseline dataset of all 1018 participants. CFA analysis was performed on three models, mirroring the original development of the TRIRISK model [17]: pooling all 18 questions into one category (single-factor model), two categories of deliberative and non-deliberative question items (two-factor model) and three categories as per the TRIRISK model (deliberative, affective and experiential). The analysis was performed using the SEM graphical user interface, choosing the maximum likelihood method and standardised factor loadings. Goodness of fit was assessed using χ2, RMSEA, SRMR and CFI. χ^2^ was interpreted in a descriptive fashion [24], as it is based on the assumption that variables are multivariate normal, and is sensitive to model complexity and large sample size [25]. RMSEA and SRMR were considered adequate if below 0.08, CFI if above 0.9 [25, 26]. A second round of CFA was performed after entering error correlations for all modification indices of >20, consistent with the methodology in the original TRIRISK publication [17]. Error correlations between measurement items crossing between different factors were omitted.

#### Results and discussion

Overall, there were very little missing data (average 1.4%, range 0% (D1)– 10.5% (D2)). The fact that 107 participants did not answer question D2 (“On a scale from 0 to 100%, how would you rate the probability that you will develop one of these five cancers in the next 10 years?”), may have been due to the unavailability of a “don’t know” option [27].

Inspecting the CFA without correlation of errors, the model fit was generally inadequate, as fit statistics such as RMSEA, SRMR and CFI were below the desired threshold (Table 2). There was an improvement going from the single to the dual factor model, but no further significant improvement going from the two- to the three-factor model. The χ^2^ statistics were high, in keeping with poor fit, but displayed the same pattern of improvement as the other fit parameters. Overall, the results indicate that there was no significant improvement in fit by splitting the non-deliberative category of risk perception of the dual model into affective and experiential factors.

**Table 2:** Fit parameters for CFA.

Parameters	Values indicative of good fit [25, 26, 28]	Without correlating errors			With error correlations*		
		Single	Dual	TRI-RISK	Single	Dual	TRI-RISK
p	p>0.05	p<0.001	p<0.001	p<0.001	p<0.001	p<0.001	p<0.001
χ^2^	low	4254	2901	2782	148	496	568
*df*	-	153	152	150	79	115	124
RMSEA	<0.08	0.190	0.156	0.154	0.041	0.070	0.072
SRMR	<0.08	0.143	0.104	0.093	0.030	0.083	0.080
CFI	>0.90	0.694	0.794	0.803	0.994	0.970	0.966

(*) 74 error correlations were entered for the single factor model and 37 for the dual model. For the TRIRISK model, 26 correlations were entered, having excluded one correlation in the affective category due to low error variance resulting in non-convergence. S1 Table 1 in S1 File shows the fit parameters for immediate follow-up to allow comparison with the baseline data shown here.

As expected, the fit parameters for CFA were better with correlation of errors (Table 2). However, these parameters are more difficult to interpret, as it is difficult to separate the effect of the different factor structures from the effect of non-comparable degrees of freedom. The fit parameters RMSEA, SRMR and CFI of the single model were within the desired ranges and generally better than those of the dual and TRIRISK models. As with the CFA there was no significant improvement in model fit by spitting the non-deliberative category into affective and experiential items.

Inspecting factor loadings of latent constructs (Table 3), we observed a wide range of magnitudes, extending from 0.13–0.94/0.13–0.96 for the CFA without and with error correlations, respectively. The factor loadings in the deliberative category (category average 0.28/0.28) were low in the single factor model, but of moderate to high magnitude in the dual and TRIRISK models (category average 0.68/67 for the dual model, mean item total correlation 0.68/0.67 for the TRIRISK model). The average factor loadings approximately doubled going from the single to the dual factor model, but did not increase further going from the dual to the TRIRISK model. Loadings of the affective category were strongest in all three models tested (category average 0.92/0.90 and 0.92/0.89, mean item total correlation 0.92/0.91 for the single, dual and TRIRISK models, respectively). Loadings of the experiential category produced the largest spread, ranging from 0.13–0.94/0.13–0.96 (E5 and E1). In the experiential category, there was no increase in the magnitude of loadings going from the single to the dual and to the TRIRISK factor model. For the dual and TRIRISK models, considering a threshold for loadings of 0.55 as defined by Falk and Miller [29], questionnaire items E3-6 are subthreshold, sharing less than 30% of the variance with the component.

**Table 3:** CFA results for the single, dual and TRIRISK model.

IDID	Question Text Question text	Standardised factor loadings					
		Without error correlations			With error correlations *		
		Single	Dual	TRI-RISK	Single	Dual	TRI-RISK
D1	How likely do you think is it that you will get one of these five cancers at some point in the next 10 years?	0.34	0.62	0.62	0.35	0.63	0.63
D2	On a scale from 0 to 100%, how would you rate the probability that you will develop one of these five cancers in the next 10 years?	0.35	0.60	0.60	0.36	0.59	0.59
D3	How do you think your chance of developing one of these five cancers in the next 10 years compares to the average person of your sex and age?	0.33	0.65	0.65	0.32	0.69	0.70
D4	The way I look after my health means that my odds of getting one of these five cancers in the future are:	0.26	0.76	0.76	0.27	0.79	0.79
D5	When I think carefully about my lifestyle, it does seem possible that I could get one of these five cancers.	0.19	0.70	0.69	0.18	0.61	0.62
D6	If I look at myself as if I was a doctor, I realise that my behaviour puts me at risk of getting one of these five cancers.	0.19	0.75	0.75	0.18	0.69	0.69
	**Category Average**	0.28	0.68	0.68	0.28	0.67	0.67
	**Mean item total correlation**	-	0.68	0.68	-	0.67	0.67
A1	How worried are you about developing cancer in the future?	0.90	0.90	0.88	0.94	0.93	0.93
A2	How fearful are you about developing cancer in the future?	0.94	0.94	0.92	0.91	0.91	0.94
A3	How nervous are you about developing cancer in your lifetime?	0.94	0.94	0.93	0.92	0.92	0.95
A4	When you think about cancer for a moment, to what extent do you feel fearful?	0.90	0.91	0.93	0.87	0.87	0.89
A5	When you think about cancer for a moment, to what extent do you feel worried?	0.91	0.92	0.94	0.87	0.87	0.89
A6	When you think about cancer for a moment, to what extent do you feel anxious?	0.90	0.90	0.92	0.86	0.85	0.87
	**Category Average**	0.92	0.92	0.92	0.90	0.89	0.91
	**Mean item total correlation**	-	-	0.92	-	-	0.91
E1	How concerned are you about developing cancer in your lifetime?	0.93	0.93	0.94	0.95	0.96	0.94
E2	How easy is it for you to imagine yourself developing cancer in the future?	0.59	0.59	0.63	0.62	0.63	0.62
E3	I feel very vulnerable to disease.	0.45	0.45	0.48	0.45	0.46	0.47
E4	I am confident that I will not get cancer.	0.42	0.41	0.46	0.44	0.43	0.46
E5	I would be lying if I said “There is no chance of me getting cancer.”	0.14	0.13	0.17	0.13	0.14	0.15
E6	My first reaction when I hear of someone getting cancer is “that is could be me someday”.	0.51	0.51	0.53	0.51	0.51	0.53
	**Category Average**	0.51	0.50	0.54	0.52	0.52	0.53
	**Mean item total correlation**	0.57	0.71	0.54	0.56	0.71	0.53

The category average represents the mean loadings within a category, when it is not treated as a factor. Mean item total correlation represents the per-factor average of loadings.

(*) 74 error correlations were entered for the single factor model and 37 for the dual model. For the TRIRISK model, 26 correlations were entered, having excluded one correlation in the affective category due to low error variance resulting in non-convergence. S1 Table 2 in S1 File shows the CFA results for immediate follow-up to allow comparison with the baseline data shown here.

### Stage 2: Exploratory factor analysis of risk perception data with cross-validation by CFA

#### Methods

EFA was performed using IBM SPSS Statistics (version 26), utilising a random half of the baseline data of the above RCT (*n* = 508), retaining the second half for subsequent confirmatory factor analysis. Sampling adequacy and sphericity were assessed by Kaiser-Meyer-Olkin measure and Bartlett’s test, respectively [30]. Factor extraction was obtained from the correlation matrix using principal axis factoring with a maximum of 100 iterations for convergence. The principal axis factoring method was used with pairwise exclusion of missing data. Initially, the maximum of 17 factors were extracted for examination, rejecting eigenvalues of less than one according to Kaiser’s criterion [31]. Multi-collinearity amongst items was assessed by examination of the correlation coefficient (r) matrix determinant and by identifying pairs of items that had r>0.8. Items with similar wording and a high correlation coefficient r>0.8 were combined, creating a new item with scores equal to the mean of the scores of the constituent items. EFA was then performed with a reduced set of items, using Varimax rotation to produce independent factors [32]. Items with a factor loading of 0.4 or above were included in that factor [33]. This ensured that only items which account for a reasonable proportion of the factor variance (16%) are included in that factor.

CFA of the EFA-based model was then performed using the second randomised half of the split dataset used for EFA (*n* = 508), using the same methods as described in Stage 1.

#### Results and discussion

The Kaiser-Meyer-Olkin (KMO) measure verified the sampling adequacy (KMO = 0.832) [30]. Bartlett’s test of sphericity yielded χ^2^ (78) = 2598.405, p<0.001, confirming the assumption of no sphericity. Inspection of the correlation coefficient (r) matrix indicated high correlation (r>0.8) among items A1-A6 and E1 (S2 Table 1 in S2 File). Taking into account the wording and meaning of the questions with high correlation, items A1, A2, A3 and E1 were combined (A1-3/E1 “How worried/fearful/nervous/concerned are you about developing cancer in the future/your lifetime”), as were items A4, A5 and A6 (A4-6 “When you think about cancer for a moment, to what extent do you feel fearful/worried/anxious?”). Despite correlation between the two combined A1-3/E1 and A4-6 items of r = 0.860 (S2 Table 2 in S2 File), the summed items were not combined further as they probed different timescales (A1-3/E1 “[…] in the future/your lifetime vs. A4-6 “[…] think about cancer for a moment”). Following Kaiser’s criterion for eigenvalues of >1, inspection of eigenvalues of the 13 remaining items confirmed that a 3-factor solution was optimal for the data (S2 Fig 1 in S2 File), accounting for 61.6% of the variance [31].

The three-factor structure derived from EFA comprised two factors of deliberative question items and one factor of a mixed subset of affective and selected experiential items (Table 4).

**Table 4:** EFA factor composition and loadings.

ID	Question Text	Factors		
		1*	2**	3***
D1	How likely do you think is it that you will get one of these five cancers at some point in the next 10 years?	0.765	-	-
D2	On a scale from 0 to 100%, how would you rate the probability that you will develop one of these five cancers in the next 10 years?	0.699	-	-
D3	How do you think your chance of developing one of these five cancers in the next 10 years compares to the average person of your sex and age?	0.525	(0.349)	-
D4	The way I look after my health means that my odds of getting one of these five cancers in the future are:	(0.460)	0.576	-
D5	When I think carefully about my lifestyle, it does seem possible that I could get one of these five cancers.	-	0.752	-
D6	If I look at myself as if I was a doctor, I realise that my behaviour puts me at risk of getting one of these five cancers.	-	0.812	-
A1-3, E1 combined	“How worried/fearful/nervous/concerned are you about developing cancer in the future/your lifetime”	-	-	0.940
A4-6 combined	“When you think about cancer for a moment, to what extent do you feel fearful/worried/anxious”	-	-	0.817
E2	How easy is it for you to imagine yourself developing cancer in the future?	(0.304)	-	0.585
E3	I feel very vulnerable to disease.	-	-	(0.371)
E4	I am confident that I will not get cancer.	(0.356)	(0.367)	(0.384)
E5	I would be lying if I said “There is no chance of me getting cancer.”	-	(0.310)	-
E6	My first reaction when I hear of someone getting cancer is “that is could be me someday”.	-	-	0.521

Values of <0.3 have been omitted in the representation for clarity. Factor loadings are sorted by size. Items were included in the factor onto which they most strongly loaded [34]. Item loadings below the threshold level of 0.4 [33] and when applying to more than one factor are shown in brackets. Naming of the EFA-based risk perception factors in subsequent sections:

(*) Factor 1: numerical-deliberative risk perception,

(**) factor 2: reflective-deliberative risk perception,

(***) factor 3: affective-experiential risk perception.

The first factor consists of the first three questions in the deliberative risk perception category (D1-D3). Examining the wording of the questions in more detail, questions D1-3 assess a more numerical understanding of risk, namely likelihood, probability and chance. This factor was therefore termed “numerical-deliberative risk perception”.

The second factor consists of questions D4-6 of the deliberative category. Questions D4-6 are more self-reflective in nature, referring to personal risk as a function of “my health”, “my lifestyle”, and “my behaviour”, respectively. The factor was therefore named “reflective-deliberative risk perception”. Question D4 refers to the “odds” of developing cancer, which also represents a numerical term similar to those in factor 1. Interestingly, question D4 also trends towards this factor, albeit with a lower loading (0.460 vs 0.576).

The third factor is composed of questions in the affective and experiential domains (“affective-experiential risk perception”). It comprises the two combined questions A1-3/E1, A4-6 and a subset of experiential questions, E2 and E6. As mentioned above, the summed questions A1-3/E1 and A4-6 are significantly correlated, and probe evoked emotions when contemplating cancer. Question E1 has also been grouped with affective items in another study [35]. Question E2 addresses the ease of imagination of developing cancer, capturing respondent’s intuition about developing the disease. Question E6 asks for the first reaction at the thought of developing cancer. It is possible that questions E2 and E6 were interpreted with a strong affective tag in respondents, resulting in the observed grouping.

Questions E3-E5 have loadings of <0.4 and were not included in any factors [34]. Question E3 was categorised along with the affective items, potentially due to the question making reference to feelings about vulnerability. Question E4 has equally low loadings across all three factors, suggesting that it was not easily interpreted by the respondents. Question E5 contains a double negative (“I would be lying if I said “There is no chance of me getting cancer.”), rendering this question more difficult to interpret.

The EFA-based model was subsequently analysed by CFA using the second randomised half of the dataset, showing a better fit than the TRIRISK model (Table 5). SRMR and CFI were within the desired ranges, whether the analysis was performed with or without error correlations. RMSEA moved in the desired range when error correlations were entered into the estimation. The χ^2^ statistic was 221 and 77 without and with error correlations, respectively, indicating a smaller disparity between the empirical and model-implied covariance matrices.

**Table 5:** Fit parameters for CFA.

Parameters	Values indicative of good fit [25, 26, 28]	This study	
		Without error correlations	With error correlations*
p	p>0.05	p<0.001	p<0.001
χ^2^	low	221	77
*df*	-	32	29
RMSEA	<0.08	0.117	0.062
SRMR	<0.08	0.075	0.037
CFI	>0.90	0.912	0.977

(*) Three error correlations were entered into the analysis.

The factor loadings observed were mostly above the desired cut-off of >0.55 (Table 6), except for E6 in the non-error correlated model [29]. The loadings obtained without error correlations were closer to those obtained by EFA. Loadings obtained without error correlations were within 0–11% of the EFA-based values, versus a 5–28% difference for the model with error correlations. The affective-experiential factor 3 displayed the widest range of factor loadings (0.50–0.99/0.57–0.81), with the combined questionnaire item A1-3/E1 (“How worried/fearful/nervous/concerned are you developing cancer in your lifetime”) possessing the highest overall loading (0.99), containing the largest amount of common variance (98%) within the factor.

**Table 6:** CFA of the EFA-based model.

IDID	Questionnaire Item	Standardised Factor Loadings	
		Without error correlations	With error correlations*
**1. Numerical-deliberative risk perception**			
D1	How likely do you think is it that you will get one of these five cancers at some point in the next 10 years?	0.77	0.64
D2	On a scale from 0 to 100%, how would you rate the probability that you will develop one of these five cancers in the next 10 years?	0.75	0.58
D3	How do you think your chance of developing one of these five cancers in the next 10 years compares to the average person of your sex and age?	0.59	0.64
	**Mean item total correlation**	0.71	0.65
**2. Reflective-deliberative risk perception**			
D4	The way I look after my health means that my odds of getting one of these five cancers in the future are:	0.65	0.85
D5	When I think carefully about my lifestyle, it does seem possible that I could get one of these five cancers.	0.81	0.60
D6	If I look at myself as if I was a doctor, I realise that my behaviour puts me at risk of getting one of these five cancers.	0.87	0.68
	**Mean item total correlation**	0.79	0.71
**3. Affective-experiential risk perception**			
A1-3, E1	“How worried/fearful/nervous/concerned are you about developing cancer in the future/your lifetime”	0.99	0.84
A4-6	“When you think about cancer for a moment, to what extent do you feel fearful/worried/anxious”	0.87	0.70
E2	How easy is it for you to imagine yourself developing cancer in the future?	0.60	0.72
E6	My first reaction when I hear of someone getting cancer is “that is could be me someday”.	0.52	0.58
	**Mean item total correlation**	0.74	0.70

Mean item total correlation represents the per-factor average of loadings.

(*) Three error correlations were entered into the analysis.

### Stage 3: Multivariable regression analysis of risk perception factors, intention to prevent cancer and self-reported behaviour change

#### Methods

To assess the predictive associations of the risk perception factors derived from exploratory factor analysis, we performed multivariable regression analysis of the three factors from Stage 2 with measures related to intention to prevent cancer and self-report measures of behaviour.

Multivariable linear regression was carried out using Stata 13 (Statacorp, Texas). The dataset comprised the intervention groups (765 subjects) after presentation with cancer risk information. Intention to prevent cancer was measured as the sum of four questions at immediate follow up after the intervention (Table 7). Intention was regressed on the EFA-based risk perception factors, adjusting for the potential confounders age and sex, study group and objective cancer risk, used as a stratification variable in the trial. Scores of questionnaire items within each risk perception factor at immediate follow-up were averaged to produce the independent variables. In addition, behaviours measured by self-report at follow-up (Table 7), were regressed on the EFA-based risk perception factors, adjusting for behaviour at baseline and the variables listed above. The assumptions for linear regression in terms of linearity, absence of multicollinearity, independence of errors, homoscedasticity and normal distribution of residuals were assessed, and minor deviations from normality for the residuals addressed by bootstrapping estimates of standard error, confidence intervals and p-values for the model coefficients. Associations were considered significant if p<0.05 and R^2^ above 0.1 [29].

**Table 7:** Measures of intention and behaviour.

ID	Question text	Scale
I1	I am determined to do everything I can to avoid getting cancer in the future.	1 = Strongly disagree to 7 = Strongly agree
I2	I am committed to engaging in behaviours that protect me against getting cancer in the future.	1 = Strongly disagree to 7 = Strongly agree
I3	I fully intend to have a lifestyle that will prevent me from getting cancer in the future.	1 = Strongly disagree to 7 = Strongly agree
I4	I will try to do all I can to avoid getting cancer in the future.	1 = Strongly disagree to 7 = Strongly agree
B1	How many units of alcohol do you drink in a typical week?	Numerical
B2	How many hours of physical activity such as brisk walking, cycling, keep fit, aerobics, swimming or jogging, do you do in a typical week?	Numerical
B3	How many portions of fruit do you eat on a typical day?	Numerical
B4	How many portions of vegetables do you eat in a typical day?	Numerical
B5	How many portions of red meat do you eat in a typical week?	Numerical
B6	How many portions of processed meat do you eat in a typical week?	Numerical

Abbreviations: I = intention, B = behaviour.

#### Results and discussion

Of the three individual factors derived from EFA in Stage 2 (Table 8), reflective-deliberative risk perception correlated negatively with intention (β = -0.949, p<0.001), whilst the affective-experiential factor correlated positively with intention (β = 1.324, p<0.001). The numerical-deliberative factor did not show a significant association with intention ((β = -0.347, p = 0.099). Associations in the former two factors are consistent with the original TRIRISK study, where separate affective and experiential factors correlated positively, whilst the single deliberative risk perception factor associated negatively with intention [17]. In that study, Ferrer *et al*. hypothesised that the negative directionality of the association of deliberative risk perception with intention was due to individuals at higher risk being less likely to engage in prevention. This may also explain the negative correlation with the reflective-deliberative factor seen in this study. The lack of association of the numerical-deliberative factor with intention in this study may be the result of the complexity of contemplating mathematical probabilities, which may fail to translate into intention [36].

**Table 8:** Multivariable regression analysis of factors of risk perception vs. intention and behaviours.

Dependent variable	F-ratio	Prob > F	R2	Regression coefficient β and p-value for EFA-based risk perception factors		
				*Numerical-deliberative*	*Reflective- deliberative*	*Affective-experiential*
**Intention**	F(7,569) = 17.34	p<0.001	0.176	−0.347 p = 0.138	−0.949 p = 0.001	1.324 p = 0.001
**Alcohol consumption**	F(8,496) = 147.5	p<0.001	0.704	−0.005 p = 0.985	−0.084 p = 0.616	0.113 p = 0.364
**Physical Exercise**	F(8,470) = 61.0	p<0.001	0.501	−0.002 p = 0.986	−0.181 p = 0.164	0.159 p = 0.095
**Fruit consumption**	F(8,500) = 66.1	p<0.001	0.514	−0.022 p = 0.638	−0.023 p = 0.735	0.121 p = 0.006
**Vegetable consumption**	F(8,500) = 59.0	p<0.001	0.486	0.027 p = 0.665	−0.051 p = 0.345	0.031 p = 0.394
**Red meat consumption**	F(8,501) = 47.1	p<0.001	0.429	0.074 p = 0.356	−0.037 p = 0.554	0.026 p = 0.622
**Processed meat consumption**	F(8,501) = 44.9	p<0.001	0.418	−0.001 p = 0.995	0.128 p = 0.058	−0.017 p = 0.708

Multivariable regression analysis of intention to change behaviour and behaviour change on EFA-based risk perception factors was adjusted for age, sex, study group and relative risk. Looking at the effects of covariates in the models, there was only one significant interaction between sex and processed meat consumption (b = 0.527, p<0.001). The regression with behaviour was additionally adjusted for baseline behaviour. As expected, there was a significant association between baseline behaviour and behaviour at follow-up (p = 0.001 for all behaviours tested). Model assumptions of linearity, independence of errors and homoscedasticity were met. The p-values for the model coefficients were calculated from bootstrapped estimates of standard errors based on 1000 bootstrapped samples. Factor compositions: Numerical-deliberative items: D1-D3, reflective-deliberative items: D4-D6, affective/experiential items: A1-3/E1, A4-6 and E2, E6.

For most of the different behaviours analysed (alcohol, red meat, fruit and vegetable consumption, physical exercise), there were no significant associations with risk perception factors, apart from two isolated pairings (affective-experiential risk perception and fruit consumption, β = 0.121, p = 0.002 and reflective-deliberative risk perception and processed meat consumption β = 0.128, p = 0.046). Given multiple testing, these measurements could potentially be due to chance. The absence of robust patterns of association are consistent with previous findings that personalised risk information in isolation does not produce strong or sustained effects on behaviour [37].

Overall, the combined regression results for intention and behaviour are consistent with the previously reported intention-behaviour gap. Whilst the cognitive processes involved in forming intentions after encounter of a threat to health are relatively well understood [38, 39], evidence suggests that intention is not necessarily linked with health behaviour change [40]. This has also been observed here, in that self-reflective and affective experiential risk perception are associated with intention, but not with subsequent behaviour change.

## General discussion and conclusions

### 1. Summary

In these linked studies, we have shown that the TRIRISK model originally developed in the US does not fit well within our UK-based dataset (Stage 1). Instead, our dataset is better characterised by a different three-factor structure in which deliberative risk perception is divided into “numerical” and “self-reflective” factors and the full set of combined affective question items is combined with a subset of experiential question items in a third factor (Stage 2). Multivariable regression showed that reflective-deliberative and affective-experiential factors (but not numerical-deliberative factors) are significantly associated with intentions to change behaviour, though there were no robust associations with behaviour change (Stage 3).

### 2. Implications for risk perception factors and their application

To our knowledge, this is the first study showing that deliberative risk perceptions may be composed of separate numerical and self-reflective components. Most previous research does not make systematic distinctions between different types of deliberative risk perceptions. In one example where a distinction between absolute and comparative components of perceived risk was made, data were subsequently combined for further analysis [41]. Distinctions within the category of deliberative risk become important when sub-categories have different predictive associations requiring them to be investigated separately, as for our findings that numerical-deliberative risk perception showed no significant association with intention, whereas reflective-deliberative risk perception associated negatively with intention. Wilson *et al*. investigated the predictive utility of probability–related risk perception with regards to intentions relating to four different hazards, resulting in mixed findings in terms of the significance and the sign of the observed associations [35]. The lack of significant association for numerical-deliberative risk perceptions in our study fits into this picture of varying predictive power of probabilistic risk perception, and could be explained by the complexity of making judgements about numerical probabilities [36].

Secondly, EFA resulted in a combined factor of all affective and a subset of experiential items. The selected experiential items either correlated with affective items (E1) or had affective connotations (E2 and E6), whilst the experiential items not included were considered less easily interpretable by the respondents (E3-E5). The resulting combined factor was strongly predictive of behavioural intentions to prevent cancer. In a previous application of the TRIRISK model, affective risk perception was found to be the most powerful predictor of protection motivation, though experiential risk perception was also a positive predictor [21].

Extrapolating the significance of the findings discussed here for the development of interventions using risk communication and the mediating role of risk perception to shape intentions, the most generalisable conclusion may be to target communications to the affective components of risk perception, as these were shown to be positive predictors of intention. By contrast, it seems less useful to target deliberative components, as these may either be negatively correlated with intention or may not have an impact on intention.

Despite showing an association with intention, this study found no robust associations between factors of risk perception and the various behaviours measured in the trial. This is not necessarily surprising given the complexity of determinants of human behaviour, including personality, attitude, subjective norms, intention, as well as environmental and other factors [42]. Any interventions targeting risk perception with an aim to change behaviour would therefore also need to attempt to optimise the effect on behaviour, for example by targeting behavioural self-regulation [43].

### 3. External validity of the TRIRISK model

In our analysis, we did not replicate the components of the TRIRISK model. Given that our research addresses risk perception from a UK perspective, this could have been due to different interpretation of the items in the TRIRISK instrument, or due to different cultural and societal perceptions of risk. Discrepancies apply in particular to UK study participants distinguishing between numerical and self-reflective perceived risk, and experiential risk perception appearing to be more closely associated with affective risk perception compared to the US. It is well established that risk is perceived differently between populations of laypeople vs. expert individuals [44], suggesting there can be substantial differences in how people conceptualise risk, which may be driven by group-based experiences, learning, or culture. Looking at inter-cultural comparisons specifically, one study found that there are cross-cultural differences in risk perception in the financial domain [45], but less evidence has been gathered about such differences in the health sector. As the study presented here was not designed to draw conclusions about country or cultural differences, a direct comparison of risk perceptions between different countries is an interesting and important topic for future research.

In addition to the possibility of different perceptions between residents of the US and UK, there were also differences in the characteristics of the participants in the two studies. The UK sample had a higher proportion of females and participants of White ethnicity compared to the US sample, and both gender and ethnicity have been linked with differences in risk perception [17, 46, 47].

Finally, in the original publication of the TRIRISK model, confirmatory factor analysis was used to verify its hypothesised factor structure with adequate fit statistics [17]. In this study, we used exploratory factor analysis to identify the underlying factor structure of our dataset, enabling the identification of a factor structure which best fits the data, which was subsequently confirmed.

### 4. Strengths and weaknesses of this study

This study represents the first application of the TRIRISK model outside the US, and one of the first times that deliberative, affective, and experiential risk perception have been examined concurrently outside the US. It benefits from a large sample size and very little missing data. We have presented confirmatory and exploratory factor analyses, in an overall approach of testing existing theory, developing a new hypothesis based on our analysis, and subsequently testing the new hypothesis. Furthermore, the study explores associations of risk perception with intentions and behaviour. A further strength is the reporting of CFA with and without the use of correlated errors, enabling assessment of the benefits of adding additional paths to the theorised model. In this study, we found that there was no benefit gained from correlating measurement errors, aside from the appearance of improved fit statistics. In fact, across-model comparison was made more difficult due to large differences in degrees of freedom, and the magnitude of the factor loadings was altered.

As the study was delivered online with computer-literate participants, results are potentially not representative of the overall UK population. The study population differed from the average UK population, in that the mean age of participants was 43.9 years (SD 10.0), with 71% females and 93% White Caucasian [23]. In the trial, behaviour was assessed by self-report, which can be inaccurate and may be affected by recall bias. In addition, this study has only addressed risk perception in relation to the single health threat of cancer.

### 5. Future work

Future work is needed to further evaluate the replicability of the two distinct deliberative risk categories in other populations, including the population used to develop the original TRIRISK model, and with regards to other illnesses. Furthermore, it would be beneficial to optimise the experiential risk perception instrument, in order to render it more generally applicable. Revisiting the wording of items addressing experiential risk perception to take into account previous experience with cancer or cancer screening, or addressing specifically those perceptions with relation to the threat that are consciously accessible may also help to better explore experiential risk [7]. It is also essential for both theoretical and practical reasons to further examine the predictive utility of the risk perception factors identified here, with regard to intentions and subsequent behaviour. As mentioned above, behaviour is itself multiply determined by factors in addition to intention, and constitutes a crucial area for on-going research.

As cancer is among the most prevalent diseases attributable to human behaviour [48], designing interventions to increase risk perception associated with these behaviours is important. Enabled by an increased understanding of how people construe risk, as derived from this study in the context of the existing literature, more effective targeting of risk perception in interventions aimed at altering cancer risk will hopefully contribute to cancer prevention and other positive health outcomes in the future.

## Supporting information

S1 File Consists of S1 Table 1—S1 Table 2. (DOCX)

S2 File Consists of S2 Table 1—S2 Table 2 & S2 Fig 1. (DOCX)
