# Supplementary material for: Analysis of the components of cancer risk perception and links with intention and behaviour: A UK-based study
Source: PLoS One. 2022 Jan 13;17(1):e0262197. doi: 10.1371/journal.pone.0262197 (PMC8757986; doi:10.1371/journal.pone.0262197)
Supplement: S1 File — (DOCX) [file pone.0262197.s001.docx]

# **Supporting Information**

## 1. Comparison of CFA at different time-points

In order to ensure that there are no differences in the results of the CFA at baseline and follow-up, the results from these two time-points have been presented side-by-side. For the baseline data, the study cohort comprised the full set of participants (1018 subjects), whilst the data at immediate follow-up comprised the intervention groups only (765 subjects)

S1 Table 1. Fit parameters for CFA at different time-points

| **Parameters** | **Values indicative of good fit** [26,27,29] | **Baseline** | | | **Immediate follow-up** | | |
| --- | --- | --- | --- | --- | --- | --- | --- |
|  |  | **Single** | **Dual** | **TRI-RISK** | **Single** | **Dual** | **TRI-RISK** |
| p | p>0.05 | p<0.001 | p<0.001 | p<0.001 | p<0.001 | p<0.001 | p<0.001 |
| χ^2^ | low | 4254 | 2901 | 2782 | 3196 | 2076 | 1995 |
| *df* | - | 135 | 134 | 132 | 135 | 134 | 132 |
| RMSEA | <0.08 | 0.190 | 0.156 | 0.154 | 0.198 | 0.158 | 0.156 |
| SRMR | <0.08 | 0.143 | 0.104 | 0.093 | 0.137 | 0.080 | 0.071 |
| CFI | >0.90 | 0.694 | 0.794 | 0.803 | 0.722 | 0.824 | 0.831 |

There is an improvement in fit statistics going from the single factor to the dual factor model for both time-points, but no further improvement in fit statistics going from the dual to the TRIRISK model. The majority of the fit statistics of the TRIRISK model are not within the desired ranges.

S1 Table 2. CFA results for the single, dual and TRIRISK Model at two time-points

| **ID** | **Question Text** | **Standardised factor loadings** | | | | | |
| --- | --- | --- | --- | --- | --- | --- | --- |
|  |  | **Baseline** | | | **Immediate follow-up** | | |
|  |  | Single | Dual | TRI-RISK | Single | Dual | TRI-RISK |
| D1 | How likely do you think is it that you will get one of these five cancers at some point in the next 10 years? | 0.34 | 0.62 | 0.62 | 0.30 | 0.62 | 0.62 |
| D2 | On a scale from 0 to 100%, how would you rate the probability that you will develop one of these five cancers in the next 10 years? | 0.35 | 0.60 | 0.60 | 0.33 | 0.60 | 0.60 |
| D3 | How do you think your chance of developing one of these five cancers in the next 10 years compares to the average person of your sex and age? | 0.33 | 0.65 | 0.65 | 0.35 | 0.79 | 0.79 |
| D4 | The way I look after my health means that my odds of getting one of these five cancers in the future are: | 0.26 | 0.76 | 0.76 | 0.32 | 0.87 | 0.86 |
| D5 | When I think carefully about my lifestyle, it does seem possible that I could get one of these five cancers. | 0.19 | 0.70 | 0.69 | 0.26 | 0.63 | 0.64 |
| D6 | If I look at myself as if I was a doctor, I realise that my behaviour puts me at risk of getting one of these five cancers. | 0.19 | 0.75 | 0.75 | 0.29 | 0.71 | 0.71 |
|  | **Category Average** | 0.28 | 0.68 | 0.68 | 0.31 | 0.70 | 0.70 |
|  | **Mean item total correlation** | - | 0.68 | 0.68 | - | 0.70 | 0.70 |
| A1 | How worried are you about developing cancer in the future? | 0.90 | 0.90 | 0.88 | 0.97 | 0.97 | 0.97 |
| A2 | How fearful are you about developing cancer in the future? | 0.94 | 0.94 | 0.92 | 0.98 | 0.98 | 0.98 |
| A3 | How nervous are you about developing cancer in your lifetime? | 0.94 | 0.94 | 0.93 | 0.97 | 0.97 | 0.97 |
| A4 | When you think about cancer for a moment, to what extent do you feel fearful? | 0.90 | 0.91 | 0.93 | 0.92 | 0.92 | 0.92 |
| A5 | When you think about cancer for a moment, to what extent do you feel worried? | 0.91 | 0.92 | 0.94 | 0.93 | 0.93 | 0.93 |
| A6 | When you think about cancer for a moment, to what extent do you feel anxious? | 0.90 | 0.90 | 0.92 | 0.91 | 0.91 | 0.91 |
|  | **Category Average** | 0.92 | 0.92 | 0.92 | 0.95 | 0.94 | 0.95 |
|  | **Mean item total correlation** | - | - | 0.92 | - | - | 0.95 |
| E1 | How concerned are you about developing cancer in your lifetime? | 0.93 | 0.93 | 0.94 | 0.93 | 0.93 | 0.95 |
| E2 | How easy is it for you to imagine yourself developing cancer in the future? | 0.59 | 0.59 | 0.63 | 0.70 | 0.70 | 0.74 |
| E3 | I feel very vulnerable to disease. | 0.45 | 0.45 | 0.48 | 0.37 | 0.36 | 0.39 |
| E4 | I am confident that I will not get cancer. | 0.42 | 0.41 | 0.46 | 0.44 | 0.43 | 0.48 |
| E5 | I would be lying if I said “There is no chance of me getting cancer.” | 0.14 | 0.13 | 0.17 | 0.08 | 0.08 | 0.12 |
| E6 | My first reaction when I hear of someone getting cancer is “that is could be me someday”. | 0.51 | 0.51 | 0.53 | 0.47 | 0.47 | 0.48 |
|  | **Category Average** | 0.51 | 0.50 | 0.54 | 0.50 | 0.50 | 0.53 |
|  | **Mean item total correlation** | 0.57 | 0.71 | 0.54 | 0.59 | 0.72 | 0.53 |

The category average represents the mean loadings within a category, when it is not treated as a factor. Mean item total correlation represents the per-factor average of loadings.
