# Supplementary material for: Analysis of the components of cancer risk perception and links with intention and behaviour: A UK-based study
Source: PLoS One. 2022 Jan 13;17(1):e0262197. doi: 10.1371/journal.pone.0262197 (PMC8757986; doi:10.1371/journal.pone.0262197)
Supplement: S2 File — (DOCX) [file pone.0262197.s002.docx]

### 2. EFA Tables and Figures

**S2 Table 1. Correlation Matrix for all original question items.**

| **ID** | D1 | D2 | D3 | D4 | D5 | D6 | A1 | A2 | A3 | A4 | A5 | A6 | E1 | E2 | E3 | E4 | E5 | E6 |
| --- | --- | --- | --- | --- | --- | --- | --- | --- | --- | --- | --- | --- | --- | --- | --- | --- | --- | --- |
| D1 | 1 |  |  |  |  |  |  |  |  |  |  |  |  |  |  |  |  |  |
| D2 | .616 | 1 |  |  |  |  |  |  |  |  |  |  |  |  |  |  |  |  |
| D3 | .445 | .406 | 1 |  |  |  |  |  |  |  |  |  |  |  |  |  |  |  |
| D4 | .459 | .427 | .552 | 1 |  |  |  |  |  |  |  |  |  |  |  |  |  |  |
| D5 | .336 | .333 | .347 | .493 | 1 |  |  |  |  |  |  |  |  |  |  |  |  |  |
| D6 | .375 | .345 | .454 | .581 | .718 | 1 |  |  |  |  |  |  |  |  |  |  |  |  |
| A1 | .349 | .365 | .283 | .261 | .190 | .188 | 1 |  |  |  |  |  |  |  |  |  |  |  |
| A2 | .323 | .341 | .301 | .231 | .162 | .191 | .913 | 1 |  |  |  |  |  |  |  |  |  |  |
| A3 | .311 | .307 | .286 | .229 | .159 | .185 | .871 | .923 | 1 |  |  |  |  |  |  |  |  |  |
| A4 | .226 | .226 | .228 | .165 | .102 | .126 | .756 | .813 | .822 | 1 |  |  |  |  |  |  |  |  |
| A5 | .241 | .245 | .243 | .182 | .129 | .145 | .765 | .811 | .832 | .943 | 1 |  |  |  |  |  |  |  |
| A6 | .235 | .262 | .245 | .174 | .123 | .138 | .741 | .791 | .823 | .912 | .942 | 1 |  |  |  |  |  |  |
| E1 | .302 | .327 | .295 | .246 | .171 | .195 | .862 | .873 | .876 | .831 | .834 | .815 | 1 |  |  |  |  |  |
| E2 | .389 | .365 | .303 | .302 | .250 | .266 | .578 | .553 | .562 | .462 | .490 | .474 | .590 | 1 |  |  |  |  |
| E3 | .330 | .291 | .321 | .308 | .220 | .242 | .403 | .393 | .391 | .354 | .361 | .354 | .416 | .370 | 1 |  |  |  |
| E4 | .437 | .375 | .368 | .393 | .385 | .318 | .401 | .378 | .379 | .291 | .315 | .310 | .400 | .491 | .383 | 1 |  |  |
| E5 | .099 | .078 | .176 | .162 | .248 | .210 | .096 | .088 | .093 | .098 | .104 | .092 | .121 | .167 | .120 | .337 | 1 |  |
| E6 | .278 | .196 | .183 | .183 | .183 | .181 | .478 | .466 | .482 | .440 | .471 | .452 | .484 | .417 | .346 | .370 | .192 | 1 |

The correlation coefficient (r) matrix determinant was very small (5.603 x 10^-8^), indicating the presence of high correlations among some observed variables in the survey. Overall, there were 17 occurrences of a correlation coefficient of r>0.8, all between the six affective questions and the first of the experiential questions (A1-6 and E1). Examination of both the r values and the wording of the questions suggested combining questions A1, A2, A3 and E1, as well as A4, A5 and A6, respectively.

**S2 Table 2.** **Correlation Matrix of original and combined question items.**

| **ID** | D1 | D2 | D3 | D4 | D5 | D6 | A1-3, E1 combined | A4-6 combined | E2 | E3 | E4 | E5 | E6 |
| --- | --- | --- | --- | --- | --- | --- | --- | --- | --- | --- | --- | --- | --- |
| D1 | 1 |  |  |  |  |  |  |  |  |  |  |  |  |
| D2 | .616 | 1 |  |  |  |  |  |  |  |  |  |  |  |
| D3 | .445 | .406 | 1 |  |  |  |  |  |  |  |  |  |  |
| D4 | .459 | .427 | .552 | 1 |  |  |  |  |  |  |  |  |  |
| D5 | .336 | .333 | .347 | .493 | 1 |  |  |  |  |  |  |  |  |
| D6 | .375 | .345 | .454 | .581 | .718 | 1 |  |  |  |  |  |  |  |
| A1-3,E1 combined | .335 | .350 | .305 | .254 | .179 | .199 | 1 |  |  |  |  |  |  |
| A4-6 combined | .239 | .251 | .246 | .178 | .122 | .140 | .860 | 1 |  |  |  |  |  |
| E2 | .389 | .365 | .303 | .302 | .250 | .266 | .597 | .486 | 1 |  |  |  |  |
| E3 | .330 | .291 | .321 | .308 | .220 | .242 | .417 | .366 | .370 | 1 |  |  |  |
| E4 | .437 | .375 | .368 | .393 | .385 | .318 | .407 | .312 | .491 | .383 | 1 |  |  |
| E5 | .099 | .078 | .176 | .162 | .248 | .210 | .105 | .106 | .167 | .120 | .337 | 1 |  |
| E6 | .278 | .196 | .183 | .183 | .183 | .181 | .500 | .465 | .417 | .346 | .370 | .192 | 1 |

The correlation coefficient (r) matrix determinant was 0.003 indicating no collinearity using the condensed variables. The single remaining correlation with r>0.8 was found between the two combined questions (“How worried/ fearful/ nervous/ concerned are you about developing cancer in the future/your lifetime” and “When you think about cancer for a moment, to what extent do you feel fearful/ worried/ anxious”, r=0.860). As these questions are probing different timescales, they were kept separate in the subsequent analysis. No items had a correlation with any other of less than 0.3 and it was likely therefore that all items could contribute to a factor.

**S2 Figure 1. Scree plot for the explanatory factor analysis of the 13-question version of the TRIRISK model.**
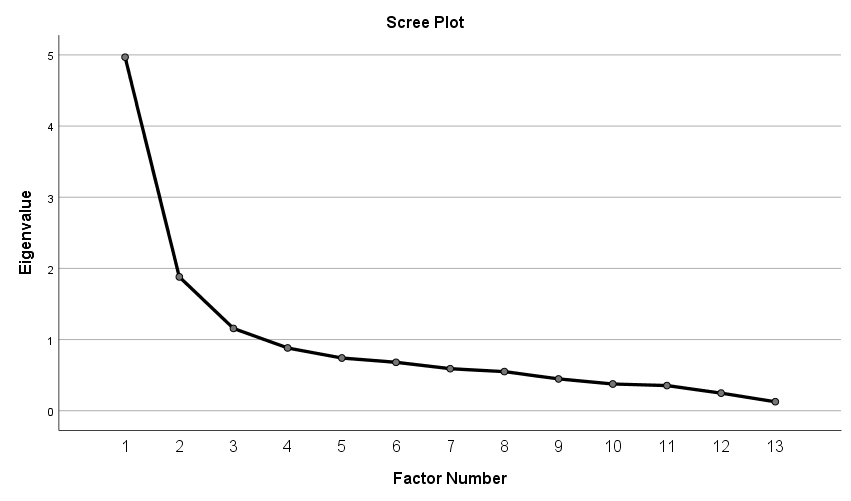


Examination of the eigenvalues and the scree plot confirmed that a 3-factor solution was optimal, explaining 61.6% of the total variance and following Kaiser’s criterion of selecting eigenvalues greater than one (32). Adding a fourth factor only explained a further 6.8% of variance. The factor analysis was subsequently repeated extracting three factors.
